# A case-control study of dietary patterns and lifestyle factors affecting colorectal cancer in Hunan province, South China

**DOI:** 10.1097/MD.0000000000050091

**Published:** 2026-08-07

**Authors:** Yanfang Qiu, Feng Yi, Rui Zhong, Caiyun Yi, Jiali Wang, Li Li, Jianghua Xie, Lei Zhu, Wei Wang, Yanhui Zou, Qiong Zhang

**Affiliations:** a Department of Radiation Oncology, Hunan Cancer Hospital/The Affiliated Cancer Hospital of Xiangya School of Medicine, Central South University, Changsha, Hunan, China; b Department of Emergency, Yueyang Central Hospital, Yueyang, Hunan, China; c Department of Medical Oncology, Digestive and Urinary Unit, Hunan Cancer Hospital/The Affiliated Cancer Hospital of Xiangya School of Medicine, Central South University, Changsha, Hunan Province, China; d Department of Nursing, Hunan Cancer Hospital/The Affiliated Cancer Hospital of Xiangya School of Medicine, Central South University, Changsha, Hunan Province, China; e Colon and Rectal Surgery Department, Hunan Cancer Hospital/The Affiliated Cancer Hospital of Xiangya School of Medicine, Central South University, Changsha, Hunan Province, China; f Gastrointestinal Surgery, Hunan Cancer Hospital/The Affiliated Cancer Hospital of Xiangya School of Medicine, Central South University, Changsha, Hunan Province, China; g Department of Otolaryngology Head and Neck Surgery, Xiangya Hospital of Central South University, Changsha, Hunan Province, China; h Department of Otolaryngology, Shenzhen People’s Hospital (The First Affliated Hospital, Southern University of Science and Technology; The Second Clinical Medical College, Jinan University), Shenzhen, China; i Hunan Cancer Hospital/The Affiliated Cancer Hospital of Xiangya School of Medicine, Central South University, Changsha, Hunan Province, China.

**Keywords:** colorectal cancer, eating preferences, influence factor, lifestyle, physical activity, sleep pattern

## Abstract

This study aimed to investigate the effects of demographic factors, diet, sleep patterns, and physical exercise on the incidence of colorectal cancer (CRC) in Hunan, China. This study consecutively recruited patients newly diagnosed with CRC between July 30, 2020, and October 27, 2021. Each case was individually matched in a 1:1 ratio with one of the patient’s family members’ data control based on gender and age (±2 years). Additionally, efforts were made to ensure that matches were closely aligned with respect to educational level, place of residence, and occupation. The results showed that participants with primary school education or below were more likely to develop CRC compared with those with college education or above (OR = 5.693, 95% confidence interval [CI] = 0.789–41.076, *P* = .085). A balanced diet appears to be a risk factor for CRC compared with a plant-preference diet (PPD) (OR = 1.726, 95%CI = 0.980–3.040, *P* = .059). The risk of CRC was 1.542 times higher in participants who drank carbonated beverages than in those who did not (OR = 1.542, 95%CI = 0.989–2.405, *P* = .056). Participants with a walking habit exhibited a protective factor relative to those without a walking habit (OR = 0.533, 95%CI = 0.392–0.725, *P* < .001). After adjusting for gender and age, our case-control study found that the following factors were associated with an increased risk of CRC: consumption of carbonated soft drinks, lack of exercise, having a balanced diet compared with a PPD, and educational attainment at primary school level or below compared with college or higher. It should be noted that people with higher education level, regular exercise routines, and walking habits can reduce their risk of CRC. However, this paired study failed to prove the influence of alcohol consumption (AC) and smoking on the incidence of CRC. Since the incidence of CRC is the result of complex effects of multiple factors, further research is needed to discover its mechanism.

## 1. Introduction

In 2020, more than 1.93 million new cases of colorectal cancer (CRC) were reported globally, accounting for 9.4% of all new cancers. The incidence of CRC is closely related to regional economic conditions.^[1]^ By 2044, the incidence and death toll of colorectal cancer in China are expected to reach 1.31 million and 484,000, respectively.^[2]^ The incidence of CRC in Hunan province is also not optimistic, with 23.91 per 100,000 new cases per year. The current incidence and mortality rate of CRC are ranked fifth and third, respectively, in Hunan Province, and show an obvious upward trend.^[3,4]^

The risk factors of CRC have long been the focus of researchers. Apart from concern with heredity as a risk factor of CRC,^[5]^ environmental factors such as dietary habits,^[6]^ obesity,^[7]^ sleep pattern,^[8]^ physical activity,^[9]^ and lifestyle^[10]^ have been studied. In addition, multiple studies have shown significant regional differences in the incidence of colorectal cancer.^[11–13]^ The results of Wang et al^[14]^ study on the risk factors of CRC in residents of Inner Mongolia located in northwest China, where people consume more meat than people in southern China. The study showed that the risk factors of CRC were an age range of 45 to 59 years, age ≥60 years old, outdoor work (mine, coal cave), a family history of CRC, and alcohol consumption (AC); being married and not smoking were also shown as protective factors for CRC. Li et al^[15]^ conducted a study on patients with colorectal cancer in Wuhan, China, and found that the risk of colorectal cancer increased among those who were divorced or widowed and those with a junior high school education or below. Obesity, smoking, AC, fatty diet, spicy diet, bacon and salted fish are also risk factors for colorectal cancer, and are in direct proportion to the amount of daily smoking and drinking time. Fruit and regular exercise are protective factors. There was also evidence of a carcinogenic effect of carbonated soft drinks on the gastrointestinal tract.^[16]^ Grain and fiber diets are protective factors for CRC.^[17]^ They are rich in fiber and low in calories, and contain phytochemicals that have antioxidant and anti-inflammatory effects.^[18]^ Hunan province is located in the south of China, where residents’ eating and drinking habits and lifestyles are markedly different from other regions. It is necessary to study the influencing factors of CRC incidence in this region to guide residents’ health literacy.

## 2. Materials and methods

### 2.1. Research setting

This hospital-based case-control study systematically enrolled patients with newly diagnosed colorectal cancer from July 30, 2020, to October 27, 2021. For each patient, a corresponding control was selected in a 1:1 family members’ data matching design, ensuring alignment based on gender and age (±2 years). Additionally, efforts were made to ensure that matches were closely aligned with respect to educational level, place of residence, and occupation. The samples were collected from various regions across Hunan Province, China. The selection of family members as the control group (CTG) was aimed at investigating the influencing factors of colorectal cancer while minimizing the impact of environmental variables.

Inclusion and exclusion criteria: Patients in the case group (CG) were ≥18 years old with CRC diagnosed by pathology (UICC 8th Edition); The CTG had never been diagnosed with CRC. The understanding and cognitive abilities of the 2 groups were normal, and participants within these 2 groups had no life-threatening or serious physical illness. This study was approved by the Ethics Committee of Hunan Cancer Hospital. The participants’ verbal consents were obtained.

### 2.2. Data collection

Researchers trained investigators to collect data on this study’s participants and to perform quality control. The participants were instructed by the investigator to fill in the electronic questionnaire with smartphones, and the elders without smartphones were instructed by the investigator to fill in a paper questionnaire. The researchers filled in the electronic form to correspond with these participants’ answers. The CG filled in the hospitalization number and verified the diagnosis of CRC in the medical record information system of the hospital. The participants in both groups reserved telephone numbers for contact to supplement when they had incomplete information or answers to questions. The data were collected, including general demographic information such as sex, habitation, age, marital status, occupation, educational level, height, weight and lifestyle habits (e.g., diet, water intake, sleep habits, fitness habits, smoking behaviors, and AC).

### 2.3. Variable definitions and criteria

1. Smoker: According to WHO (1998), a smoker refers to a person who has smoked continuously or cumulatively for 6 months or more in their life.^[19]^In this study, both former and current smokers were statistically analyzed.
2. Drinking: not limited to drinking type ≥once a week, continuous drinking ≥1 year. Abstainers and current drinkers were statistically analyzed. Drink wine kind all convert into white wine to calculate drink alcohol quantity.
3. Dietary habits: To capture overarching dietary patterns relevant to the study population, we employed a categorical classification method. Participants were asked to self-identify their usual long-term dietary pattern into one of 3 mutually exclusive categories, which were designed to reflect broad, conscious dietary choices and align with commonly observed eating habits in Hunan Province: Meat-preference diet (MPD): characterized by a self-reported conscious choice to have meat as the central component of most meals; PPD: characterized by a self-reported conscious choice to have plant-based foods as the central component of most meals, with minimal meat; and balanced diet: for those who did not identifying strongly with either of the above patterns, with a roughly equal emphasis on plant and animal sources. This approach aims to classify individuals based on their overall dietary orientation.
4. Drinking habits: Hot water was defined as higher than the oral temperature, warm water was defined as roughly the same as the oral temperature, cold water was defined as lower than the oral temperature, and ice water was defined as adding ice to cold water.
5. Physical Activity: Physical activity was assessed using a two-step questionnaire. First, participants were asked to report their general exercise frequency by selecting one of the following 4 categories: “Everyday,” “≥1 times per week,” “Occasionally,” and “None.” Subsequently, participants who selected any frequency other than “No exercise” were asked to specify their primary types of exercise from a list that included options such as walking, running, yoga, swimming, and others. For participants who chose any frequency other than “no exercise,” they were required to specify their main type of exercise.

### 2.4. Statistical analysis

Electronic questionnaire results were exported to Excel and SPSS (Version 25.0; SPSS, Chicago). Cross-tables were used to analyze data and frequencies, and percentages were used to describe. Chi-square test was used to compare the ratio or constituent ratio of 2 more groups. Univariate conditional logistic regression analysis and multivariate conditional logistic regression analysis were performed by including statistically significant items into the equation. αin = 0.05, αout = 0.1. Odds ratio (ORS) and 95% confidence interval (CI) calculations were evaluated using multivariate analysis. The statistical significance was *P* < .05. Since this study aims to explore the potential risk factors for colorectal cancer in this region, some of the analyses are exploratory. To minimize Type II errors, we did not perform multiple test corrections on the *P*-values in our analysis. Therefore, for the critically significant results presented in the report, their interpretation must be extremely cautious. It is necessary to consider them in conjunction with the effect size and biological plausibility for a comprehensive assessment.

## 3. Results

### 3.1. Characteristics of paired and demographic data of CG and CTG

The researchers collected a total of 203 cases in CG, nine of which with incomplete information were excluded. Among the 194 valid cases in CG, 4 cases were not successfully matched because CTG did not meet the age-matching conditions. In CTG, 1150 questionnaires were collected, and 31 questionnaires could not be supplemented due to incomplete deletion of the data. Finally, the 2 groups were the same with regard to gender, age was matched according to age ±2 years. One hundred ninety pairs of participants were matched 1:1.

CG males were slightly higher than females (54.7% vs 45.3%), and the median age was 58.00 years (18–85 years). Most of the participants were in the age range of 40 to 65 years of age (115/190, 60.6%). Those living in rural areas (60.5%) were significantly higher than those living in urban areas (39.5%). Most of the participants were retired or homestays, accounting for 45.8% (87/190) of participants.

No significant differences were found in gender, age, habitation and occupation between the 2 groups (*P* > .05, Table 1). 66.8% of CG had a low level of primary education or below. The mean body mass index (BMI) of the 2 groups was 22.49 (14.7–31.2), and a BMI of 18.5 to 23.9 was the majority in both groups, approximately accounting for 57% of participants. Obesity was 4.7% vs12.6%, respectively. Participants who were underweight were 11.1% vs6.3% in CG vs CTG, respectively. No statistically significant differences were seen in marital status, education level and BMI of the 2 groups (*P* < .05), Table 1.

**Table 1:** Univariate analysis of lifestyle between colorectal cancer patients and matched groups.

Variables	Total	Control		Case		*χ* ^2^	*P*
	N = 380 (%)	N = 190	%	N = 190	%		
Gender						0.000	1.000
Male	208 (54.7)	104	54.7	104	54.7		
Female	172 (45.3)	86	45.3	86	45.3		
Age (yr)						0.117	.943
18–39	34 (8.9)	17	8.9	17	8.9		
40–65	229 (60.3)	114	60.0	115	60.6		
≥65	117 (30.8)	59	31.1	58	30.5		
Habitation						0.876	.349
City	159 (41.8)	84	44.2	75	39.5		
Rural	221 (58.2)	106	55.8	115	60.5		
Marriage						–	.030*
Unmarried	13 (3.4)	7	3.7	6	3.2		
Married	346 (91.1)	179	94.2	167	87.9		
Divorced	6 (1.6)	1	0.5	5	2.6		
Widow	15 (3.9)	3	1.6	12	6.3		
Education						40.146	<.001
Primary school/under	195 (51.3)	68	35.8	127	66.8		
Middle school	116 (30.5)	77	40.5	39	20.5		
High school	58 (15.3)	35	18.4	23	12.1		
College or above	11 (2.9)	10	5.3	1	0.6		
Occupation						5.235	.284
Farmer	84 (22.1)	36	18.9	48	25.3		
Worker	54 (14.2)	24	12.6	30	15.8		
Self-employed	34 (8.9)	21	11.2	13	6.8		
Retired/homestay	180 (47.4)	93	48.9	87	45.8		
Unemployed	28 (7.4)	16	8.4	12	6.3		
BMI						9.958	.019
<18.5	33 (8.7)	12	6.3	21	11.1		
18.5–23.9	218 (57.3)	110	57.9	108	56.8		
24.0–28.0	96 (25.3)	44	23.2	52	27.4		
>28	33 (8.7)	24	12.6	9	4.7		
Eating						6.592	.037
Meat-preference diet	24 (6.3)	14	7.4	10	5.3		
Balanced diet	316 (83.2)	149	78.4	167	87.9		
Plant-preference diet	40 (10.5)	27	14.2	13	6.8		
Eating breakfast						-	.831*
Every day	302 (79.5)	148	77.9	154	81.1		
Regularly	62 (16.3)	33	17.4	29	15.3		
Occasionally	8 (2.1)	5	2.6	3	1.6		
Never	8 (2.1)	4	2.1	4	2.0		
Midnight snack						-	.695*
Every day	7 (1.8)	4	2.1	3	1.6		
Regularly	6 (1.6)	4	2.1	2	1.1		
Occasionally	124 (32.7)	58	30.5	66	34.7		
Never	243 (63.9)	124	65.3	119	62.6		
Hot water						1.678	.195
Yes	74 (19.5)	32	16.8	42	22.1		
No	306 (80.5)	158	83.2	148	77.9		
Warm water						0.293	.588
Yes	251 (66.1)	128	67.4	123	64.7		
No	129 (33.9)	62	64.7	67	35.3		
Cold water						4.380	.036
Yes	85 (22.4)	34	17.9	51	26.8		
No	295 (77.6)	156	82.1	139	73.2		
Iced water						1.821	.177†
Yes	9 (2.4)	2	1.1	7	3.7		
No	371 (97.6)	188	98.9	183	96.3		
Chinese tea						0.011	.917
Yes	159 (41.8)	79	41.6	80	42.1		
No	221 (58.2)	111	58.4	110	57.9		
Carbonated drinks						14.393	<.001
Yes	27 (7.1)	4	2.1	23	12.1		
No	353 (92.9)	186	97.9	167	87.9		
Fruit juice						2.098	.147
Yes	25 (6.6)	9	4.7	16	8.4		
No	355 (93.4)	181	95.3	174	91.6		
Functional beverage						4.746	.029
Yes	14 (3.7)	3	1.6	11	5.8		
No	366 (96.3)	187	98.4	179	94.2		
Milk tea						0.822	.364
Yes	20 (5.3)	8	4.2	12	6.3		
No	359 (94.7)	181	95.8	178	93.7		
Coffee						1.991	.158
Yes	13 (3.4)	4	2.1	9	4.7		
No	367 (96.6)	186	97.9	181	95.3		
Smoking						1.118	.290
Yes	144 (37.9)	67	35.3	77	40.5		
No	236 (62.1)	123	64.7	113	59.5		
Alcohol consumption						0.356	.551
Yes	93 (24.5)	44	23.2	49	25.8		
No	287 (75.5)	146	76.8	141	74.2		
Exercise frequency						6.470	.091
Everyday	126 (33.2)	62	32.6	64	33.7		
≥1 times per wk	38 (10.0)	18	9.5	20	10.5		
Occasionally	144 (37.9)	82	43.2	62	32.6		
None	72 (18.9)	28	14.7	44	23.2		
Exercise-Walk						44.475	<.001
Yes	189 (49.7)	127	66.8	62	32.6		
No	191 (50.3)	63	33.2	128	67.4		
Exercise-run						7.274	.007
Yes	86 (22.6)	32	16.8	54	28.4		
No	294 (77.4)	158	83.2	136	71.6		
Exercise-swimming						0.000	1.000†
Yes	1 (0.3)	0	0.0	1	0.5		
No	379 (99.7)	190	100.0	189	99.5		
Exercise-fitness						0.080	.778
Yes	13 (3.4)	7	3.7	6	3.2		
No	367 (96.6)	183	96.3	184	96.8		
Dance aerobics						0.269	0.604
Yes	37 (9.7)	17	8.9	20	10.5		
No	343 (90.3)	173	91.1	170	89.5		
Exercise-yoga						0.000	1.000†
Yes	7 (1.8)	3	1.6	4	2.1		
No	373 (98.2)	187	98.4	186	97.8		
Sleep pattern						2.148	.709
Before 20:00	27 (7.1)	10	5.3	17	9.0		
20:00–22:00	147 (38.7)	77	40.5	70	36.8		
22:00–24:00	164 (43.2)	82	43.2	82	43.2		
After 24:00	24 (6.3)	12	6.3	12	6.3		
Irregular	18 (4.7)	9	4.7	9	4.7		
Daily sleep time						2.199	.532
<4hr	10 (2.6)	5	2.6	5	2.6		
4–6hr	73 (19.2)	40	21.1	33	17.4		
6–8hr	220 (57.9)	103	54.2	117	61.6		
>8hr	77 (20.3)	42	22.1	35	18.4		
Insomnia frequency						4.779	.189
≥3times per wk	26 (6.8)	9	4.7	17	8.9		
1–2 times per wk	31 (8.2)	18	9.5	13	6.8		
Occasionally	232 (61.1)	122	64.2	110	57.9		
Never	91 (23.9)	41	21.6	50	26.4		
Stay up late						0.144	.705
Yes	79 (20.8)	38	20.0	41	21.6		
No	301 (79.2)	152	80.0	149	78.4		
Stay up late frequency						1.697	.638
≥3 times per wk	24 (6.3)	10	5.3	14	7.4		
1–2 times per wk	22 (5.8)	13	6.8	9	4.7		
Occasionally	33 (8.7)	15	7.9	18	9.5		
Never	301 (79.2)	152	80.0	149	78.4		

* Monte Carlo *P* < .05.

† Correction for continuity.

BMI = body mass index.

### 3.2. Eating and drinking habits

Most of the participants in CG of this study had a balanced diet (BD), accounting for 87.9% (167/190), and few of them had MPD and PPD. The participants in CG and CTG had a BD (78.4% vs 87.9%) and PPD (14.2% vs 6.8%). No difference was shown in the 2 groups of MPD (7.4% vs 5.3%) (*P* = .037).

**Figure 1.**
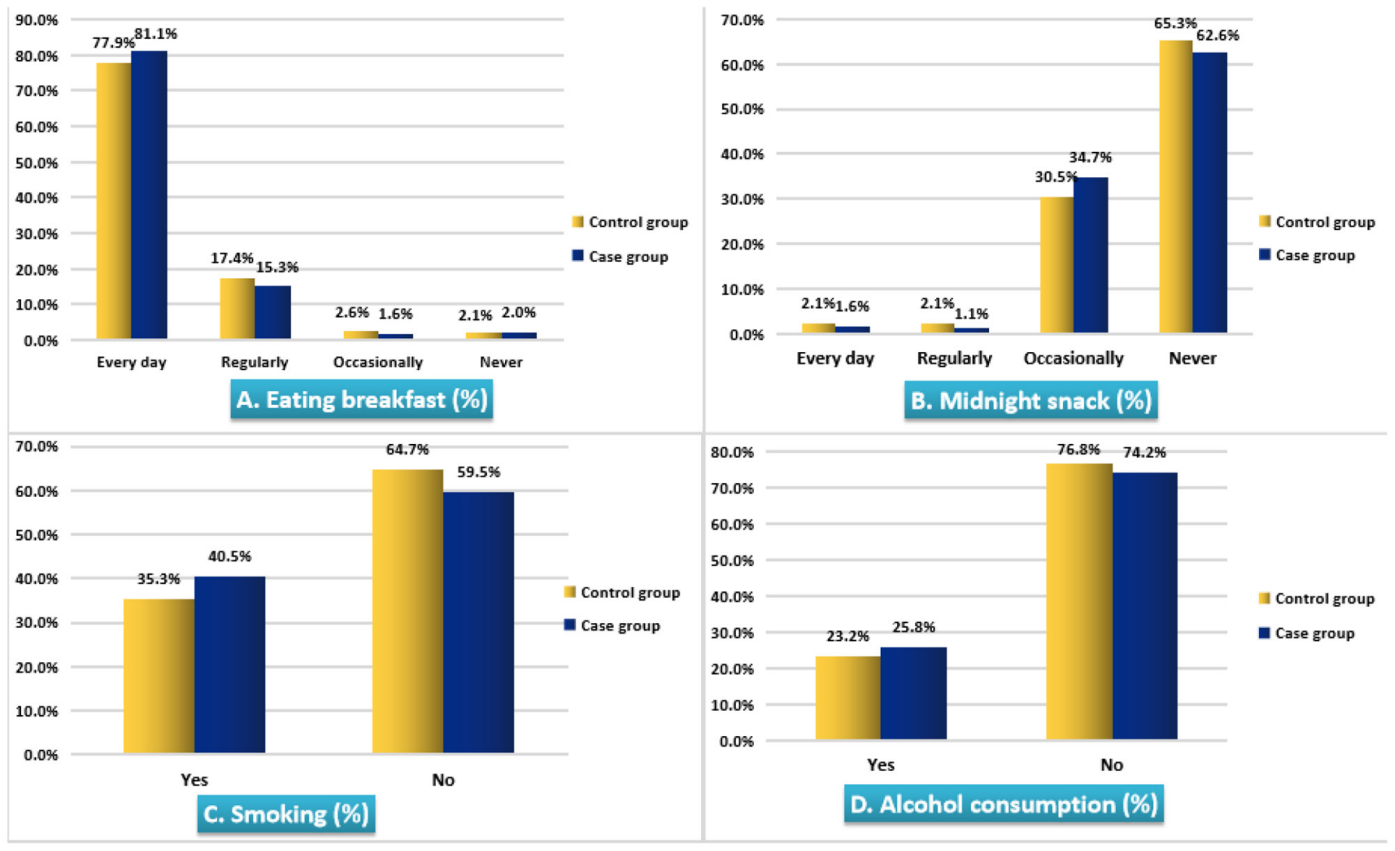
(A) Eating breakfast represent the proportional distribution characteristics of the eating breakfast of the control and case groups; (B) Midnight snack represent the proportional distribution characteristics of the midnight snack of the control and case groups; (C) Smoking represents the proportional distribution characteristics of members of the control group and the case group with smoking behavior; (D) Alcohol consumption represents the proportional distribution characteristics of members of the control group and the case group with drinking behavior.

The majority of participants in both groups ate breakfast daily (81.1% in CG vs 77.9% in CTG), as shown in Figure 1–A. Most of the study participants in both groups did not have the habit of eating midnight snacks, and only 1.8% of them ate midnight snacks every day, as shown in Figure 1–B.

Results of drinking habits showed that the proportion of cold water drinkers in CG (17.9%) was lower than that in CTG (26.8%) (*P* = .036). The number of regular tea drinkers in both groups was about 42.0%. The participants in CG drank carbonated drinks and functional drinks at a slightly higher rate than CTG (12.1% vs 2.1% and 5.8% vs 1.6%, respectively, *P* < .05), Table 1.

### 3.3. The participants’ smoking and drinking habits

The smoking rate in CG was slightly higher than that in CTG (40.5% vs 35.3%, *P* = .29), as shown in Figure 1–C. There was no statistically significant difference in drinking rate between the 2 groups (*P* = .375), as shown in Figure 1–D.

### 3.4. Physical activity

Among all the participants included in this study, 80.5% (306/380) had the habit of exercising. Among the 6 common daily exercises, participants in CG and CTG seldom chose swimming and yoga, accounting for only 1.8% (7/380) and 0.3% (1/380) of participants, respectively.

The walking physical activity of CG was (32.6%, 62/190) and significantly lower than that of CTG (66.8 %, 128/190). The preference for running for physical activity in CG (28.4%, 54/190) was slightly higher than that in CTG (16.8%, 32/190), with statistical significance (*P* < .05).

### 3.5. Sleep patterns

**Figure 2.**
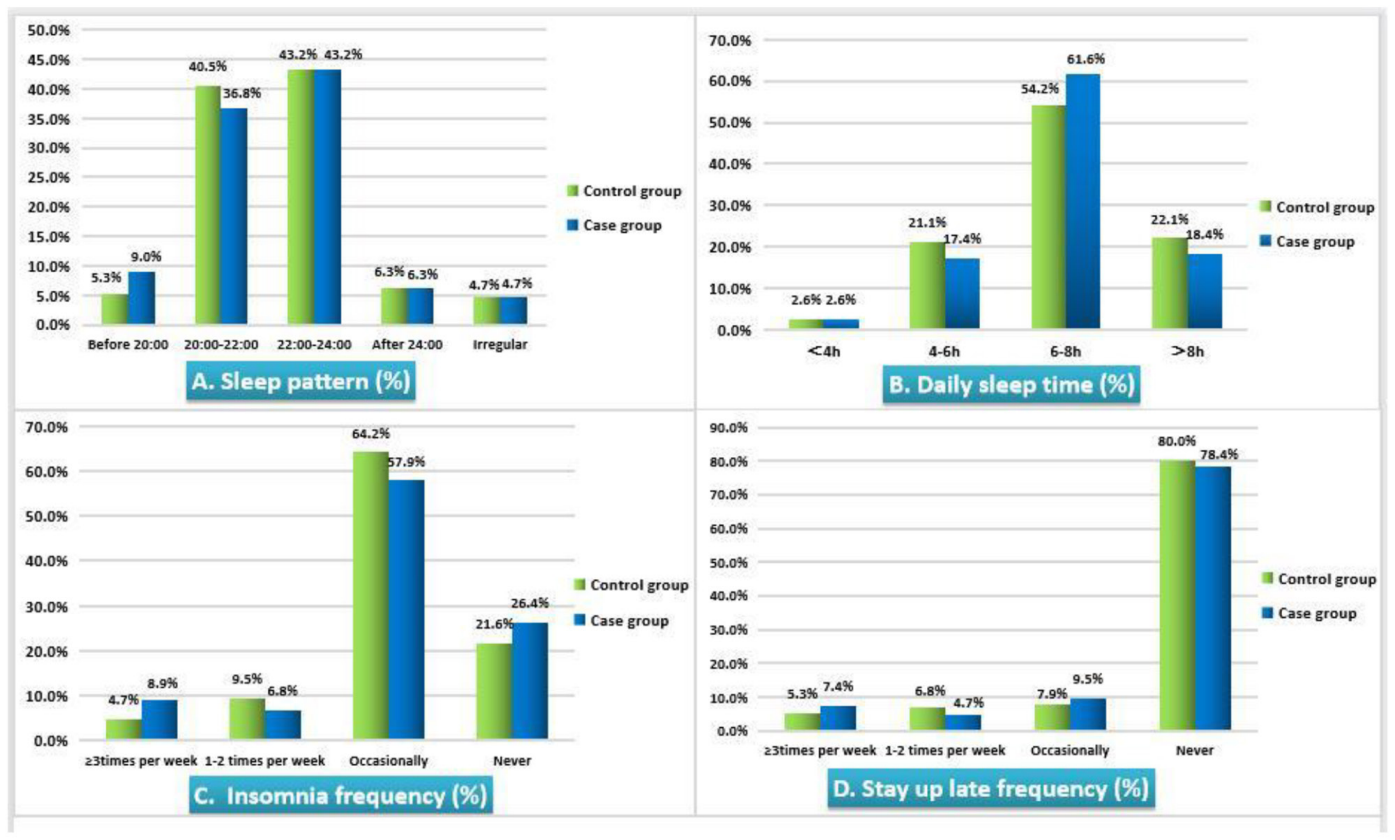
(A) Sleep pattern represent the proportional distribution characteristics of the sleep pattern of the control and case groups; (B) Daily sleep time represent the proportional distribution characteristics of the daily sleep time of the control and case groups; (C) Insomnia frequency represents the proportional distribution characteristics of the Insomnia frequency of the control and case groups; (D) Stay up late frequency represents the proportional distribution characteristics of the stay up late frequency of the control and case groups.

The 2 groups of participants in this sample maintained good sleep patterns. There were no significant differences in time to fall asleep, time to go to bed, insomnia and staying up late (*P* > .05). Most of the participants (81.9%) had a habit of falling asleep between 20:00 and 24:00. Fewer of them chose to fall asleep before 20:00 and after 24:00, accounting for 7.1% and 6.3% of participants, respectively, and 4.7% had irregular sleep patterns. More than half of the participants slept 6 to 8 hours a day, and only 2.6 % slept <4 hours. More than half of the participants (61.1%) experienced occasional insomnia, while 6.8% had insomnia frequency ≥3times per week. In addition, only 20.8% of the participants had a history of staying up late, and 6.3% had a frequency of staying up late ≥3times per week, as shown in Figure 2 (A–D).

### 3.6. Multivariate conditional logistic regression analysis

The risk of CRC was taken as the dependent variable, and the differences in marital status, education level, BMI group, eating habits, cold water intake, carbonated drinks consumption, functional drinks consumption, and running and walking were analyzed by univariate conditional logistic regression. The results showed that the overall trend of CRC incidence decreased with the increase of education level, and that participants with primary school education or below were more likely to develop CRC than those with college education or above (OR = 5.693, 95%CI = 0.789–41.076, *P* = .085). A BD appears to be a risk factor for CRC compared with a PPD (OR = 1.726, 95%CI = 0.980–3.040, *P* = .059).

The risk of CRC was 1.542 times higher in those who drank carbonated beverages than in those who did not (OR = 1.542, 95%CI = 0.989–2.405, *P* = .056). Those with a walking habit were a protective factor relative to those without a walking activity (OR = 0.533, 95%CI = 0.392–0.725, *P*<.001). Table 2.

**Table 2:** Univariate analysis and multivariate analysis of conditional logistic regression in case and control groups.

Variables	Univariate analysis OR (95%CI)	*P*	Multivariate analysis OR (95%CI)	*P*
Marriage				
Unmarried	0.577 (0.217–1.537)	0.271		
Married	0.603 (0.336–1.084)	0.091		
Divorced	1.042 (0.367–2.957)	0.939		
Widow	1			
Education				
Primary school/under	7.164 (1.001–51.252)	0.050	5.693 (0.789–41.076)	.085
Middle school	3.698 (0.508–26.918)	0.197	3.249 (0.442–23.859)	.247
High school	4.362 (0.589–32.300)	0.149	3.813 (0.512–28.417)	.192
College or above	1		1	
Occupation				
Farmer	1			
Worker	0.764 (0.443–1.319)	0.334		
Self-employed	0.606 (0.339–1.082)	0.091		
Retired	0.620 (0.43–0.891)	0.010		
Unemployed	0.699 (0.383, 1.275)	0.243		
BMI				
<18.5	2.333 (1.069–5.095)	0.033		
18.5–23.9	1.817 (0.920–3.586)	0.085		
24.0–28.0	1.986 (0.979–4.030)	0.057		
>28	1			
Eating habits				
Meat-preference diet	1.282 (0.562–2.924)	0.555	1.183 (0.516–2.711)	.692
Balance diet	1.626 (0.925–2.859)	0.091	1.726 (0.980–3.040)	.059
Plant-preference diet	1		1	
Cold water				
No	1			
Yes	1.273 (0.924–1.755)	0.140		
Carbonated drinks				
No	1		1	
Yes	1.801 (1.164–2.784)	0.008	1.542 (0.989–2.405)	.056
Functional beverage				
No	1			
Yes	1.607 (0.874–2.953)	0.127		
Run				
No	1			
Yes	1.357 (0.990–1.860)	0.057		
Walk				
No	1		1	
Yes	0.490 (0.361–0.663)	0.000	0.533 (0.392–0.725)	<.001

* Monte Carlo *P* < .05.

BMI = body mass index, OR = odd-ratio, 95%CI = 95% confidence interval.

## 4. Discussion

### 4.1. Lower level of education is a risk factor for CRC

Our study shows that people with lower education have a high risk of CRC, and that education level is a protective factor against CRC, which is consistent with the results of Imad et al^[20]^ This might be because higher educational levels are usually associated with better health knowledge, better access to medical care, and healthier lifestyles. The study failed to achieve strong significance, which might be related to the relatively limited sample size or the population specificity. Wang et al^[21]^ conducted a cross-sectional survey among 6668 people aged 20 to 65 in Guangzhou, China, and found that adults with higher education levels had higher cognition levels and more positive attitudes toward the risk factors and signs of CRC. Winterich et al^[22]^ also showed that with an increase in education level, men’s awareness of CRC also increased. CRC is a preventable disease, and most CRC can be prevented by colonoscopy.^[23]^ When people perceive a serious threat, and there are ways to reduce it, they are more likely to take action to reduce the threat.^[24]^ People with more education were shown to be more likely to voluntarily attend cancer screening than those with less education. Screening and monitoring can reduce the risk of CRC by identifying and removing precancerous lesions. Juon et al^[25]^ found that people with a high education level have a high level of CRC knowledge, and CRC knowledge level has a strong predictive effect on CRC screening behavior. In addition, highly educated people are more likely to have higher economic income and better access to medical resources; they are also more likely to accept new medical ideas to change their lifestyles and associated risk of cancer.^[26,27]^ The risk of CRC is higher in people with a lower education level, which may be related to a lack of knowledge about CRC, failure to follow the relevant lifestyle guidelines, and failure to undergo colonoscopy screening.

### 4.2. Walking has been identified as a significant protective factor against CRC

Regular physical activity can improve basic metabolism and improve tissue oxygenation, so that the body can obtain better metabolic efficiency and ability. Animal studies^[28]^ have shown that exercise can change the composition of gut microbes and increase the production of short-chain fatty acids, thereby inhibiting cancer cells. In addition, physical activity can prevent CRC by reducing inflammation as well as lowering insulin levels and insulin resistance.^[29]^ The study^[30]^ showed that more than 30 minutes of exercise per day was associated with an 11% reduction in CRC risk. Television viewing time, occupational sitting time, and total sitting time were all positively associated with CRC risk. An increase of 2 hours per day in total sitting time was associated with a 2% increase in CRC risk (RR 1.02, 95% CI 1.01–1.06).^[31]^ The results of Zhang et al^[32]^ showed that any exercise can reduce the incidence of CRC regardless of the individual’s body weight or body fat distribution. Moreover, walking is the simplest, most economical, effective, and suitable exercise for this population. For a long time, walking was more of a casual activity for leisure. With the development of society, the important value of walking has received increased attention in the medical field, and even September 29 is annually designated as “World Walking Day.” In this study, 66.8% (127/190) of the control participants used walking as a form of exercise. This finding is consistent with previous studies, showing that walking can act as a protective factor to reduce the risk of CRC.^[33,34]^ Further, the result suggests that exercise improves the body’s defence against cancer.

### 4.3. A balanced diet associated with a higher risk of CRC than a plant-based diet

The results of multivariate conditional logistic regression analysis indicated that, compared to a preference for a plant-based diet, a balanced diet was associated with a higher risk of CRC (*P* = .059). This finding underscores the potential protective role of diets rich in plant-based foods, which is consistent with the results of Orlich et al^[35]^ High dietary fiber intake, a hallmark of PPDs, is fermented by gut microbiota to produce short-chain fatty acids like butyrate. These metabolites exert anticarcinogenic effects through multiple pathways, including inducing apoptosis of cancer cells, inhibiting proliferation, and modulating anti-inflammatory and antioxidant responses.^[36–38]^ Furthermore, dietary fiber accelerates intestinal transit, thereby reducing the exposure time of the colonic mucosa to potential carcinogens.^[39]^ This biological plausibility is supported by intervention studies, such as the dietary exchange between African Americans and rural Africans, which demonstrated that a high-fiber, low-fat diet can rapidly alter gut microbiota and mucosal biomarkers toward a profile associated with reduced cancer risk.^[40]^ However, it should be clarified that “balanced diet” here is used as a comparative category, and the increased risk is in comparison to a strict plant-based diet. This might imply that, in the population studied in this research, even a diet that is considered “balanced” could have an intake of animal-based foods or processed foods that is sufficient to increase the risk of CRC.

This leads to a critical discussion of regional dietary specificity. Our study is contextualized within Hunan’s distinct food culture, which is renowned for its high consumption of preserved foods, such as cured meats (e.g., larou). It is particularly noteworthy to discuss the potential mechanisms linking these preserved foods to CRC risk, even though our broad categorization of “meat consumption” did not yield a significant association. These foods are established sources of potential carcinogens: First, processed and preserved meats are known to contain N-nitroso compounds (NOCs) and polycyclic aromatic hydrocarbons (PAHs), which are genotoxic and can directly damage colonic epithelial DNA.^[41,42]^ Second, the high salt content in these preserved products can damage the gastric and colonic mucosa, increasing susceptibility to carcinogens and potentially promoting inflammatory responses.^[43]^

The null finding for general meat intake in our study highlights the limitation of broad dietary categories and underscores the importance of examining specific food processing methods. The discrepancy between our results and the established literature may be attributed to several region-specific factors: the universal high consumption of preserved meats across both cases and controls in Hunan may have minimized the contrast in exposure, attenuating the observable effect; the local diet is also rich in potentially protective ingredients such as chili peppers and other spices, which may counteract the detrimental effects of processed meats through their antioxidant and anti-inflammatory properties^[44]^; and long-term dietary habits may induce adaptations in the gut microbiome of the local population, potentially altering the metabolic processing of these carcinogens.^[45]^ Future research could employ more precise quantitative assessments and integrate microbiome profiling to further elucidate the complex interplay between local food culture and CRC risk.

### 4.4. Drinking carbonated beverages is a risk factor for CRC

The results of multivariate conditional logistic regression analysis indicated a significant association between carbonated beverage consumption and an increased risk of CRC, with drinkers exhibiting 1.542 times higher odds compared to nondrinkers (OR = 1.542, 95% CI = 0.989–2.405, *P* = .056). The widespread consumption of carbonated beverages has raised public health concerns due to their possible adverse effects. Several pathways may explain their potential role in carcinogenesis. First, the high sugar content in many carbonated drinks can contribute to hyperinsulinemia and insulin resistance, activating the insulin-like growth factor pathway, which promotes cell proliferation and inhibits apoptosis in colonic epithelium.^[46]^ Second, carbon dioxide itself may induce gastrointestinal discomfort, mucosal irritation, or altered gut motility, though direct human evidence remains limited.^[17]^ Additionally, chemical constituents such as 4-methylimidazole, a compound found in caramel colouring used in many colas, have been debated for their dual role; some experimental studies suggest concentration-dependent antitumor effects,^[47]^ while others raise concerns about possible carcinogenicity in high exposures.^[48]^

Whether drinking carbonated beverages increases the risk of CRC remains inconsistent. A meta-analysis by Zhang et al^[49]^ of 13 prospective cohorts did not find a significant association between high intake of sweetened carbonated soft drinks and colon cancer risk. In contrast, other studies, including one by Li et al^[50]^ have reported positive correlations between sugar-sweetened beverages and cancers including CRC. These discrepancies may arise from variations in study design, population characteristics, consumption patterns, and adjustment for confounders. Notably, in the dietary context of Hunan, where diets are often high in salt, preserved meats, and spices, the additional load of carbonated beverages may exacerbate metabolic and inflammatory pathways, although this interaction requires further investigation.

However, this study did not find a correlation between smoking and AC and CRC risk, which is consistent with the findings of other studies.^[51]^ Although the results of this study showed that smoking and drinking were not associated with CRC risk, smoking and drinking have been shown to be risk factors for a number of diseases receiving worldwide attention. Therefore, not smoking and limiting AC is still a healthy lifestyle. In addition, the relationship between BMI and the risk of CRC was not significant either, which might be related to BMI values were collected after diagnosis of colorectal cancer in the CG. The weight loss caused by the tumor itself or its treatment may have weakened the true association between BMI and the risk of CRC.

### 4.5. Limitation

This study has certain limitations. Firstly, the matched controls were selected from a family database of newly diagnosed colorectal cancer patients. While the use of family members as controls allows for partial matching of shared living environments and socioeconomic factors, it may introduce selection bias. This bias arises because family members often share similar lifestyle habits, dietary patterns, and environmental exposures, all of which may be associated with colorectal cancer risk. Consequently, this matching design could underestimate the effects of some environmental or lifestyle-related risk factors. Secondly, the assessment of dietary patterns relied on a broad self-categorization. While this method efficiently captures an individual’s perceived overall dietary orientation, it is susceptible to recall bias and non-differential misclassification. Additionally, the measurement of physical activity, while capturing overall exercise frequency and specific types, lacked details on the duration of each exercise session and the precise intensity within each activity type. This limitation prevents the calculation of total energy expenditure and may hinder a fine-grained dose-response analysis and direct comparability with studies that employ more granular quantitative measures.

## 5. Conclusions

In this case-control study, higher levels of education, a diet rich in fruits and vegetables, regular walking and less frequent consumption of carbonated beverages reduced the risk of colorectal cancer. The study was not able to prove the effect of AC, smoking and BMI on colorectal cancer rates. Whether this is the result of the comprehensive effects of dietary habits and CRC incidence in southern China remains to be further studied.

## Acknowledgments

We would like to thank Yu RH, a statistical experts of School of Public Health, Central South University guided the data analysis in this study. We would like to thank Editage (www.editage.com) for English language editing.

## Author contributions

**Conceptualization:** Yanfang Qiu.

**Funding acquisition:** Yanfang Qiu, Yanhui Zou.

**Writing: original draft:** Yanfang Qiu, Feng Yi, Rui Zhong, Qiong Zhang.

**Writing: review & editing:** Yanfang Qiu, Feng Yi, Rui Zhong, Yanhui Zou, Qiong Zhang.

**Data curation:** Feng Yi, Caiyun Yi, Jiali Wang, Li Li, Jianghua Xie, Lei Zhu, Qiong Zhang.

**Investigation:** Feng Yi, Caiyun Yi, Jiali Wang, Li Li.

**Methodology:** Rui Zhong.

**Formal analysis:** Jianghua Xie, Lei Zhu.

**Project administration:** Wei Wang, Yanhui Zou.

**Supervision:** Wei Wang, Yanhui Zou.
